# Supplementary figures and images for: Gametic specialization of centromeric histone paralogs in Drosophila virilis
Source: Life Sci Alliance. 2021 May 13;4(7):e202000992. doi: 10.26508/lsa.202000992 (PMC8200288; doi:10.26508/lsa.202000992)

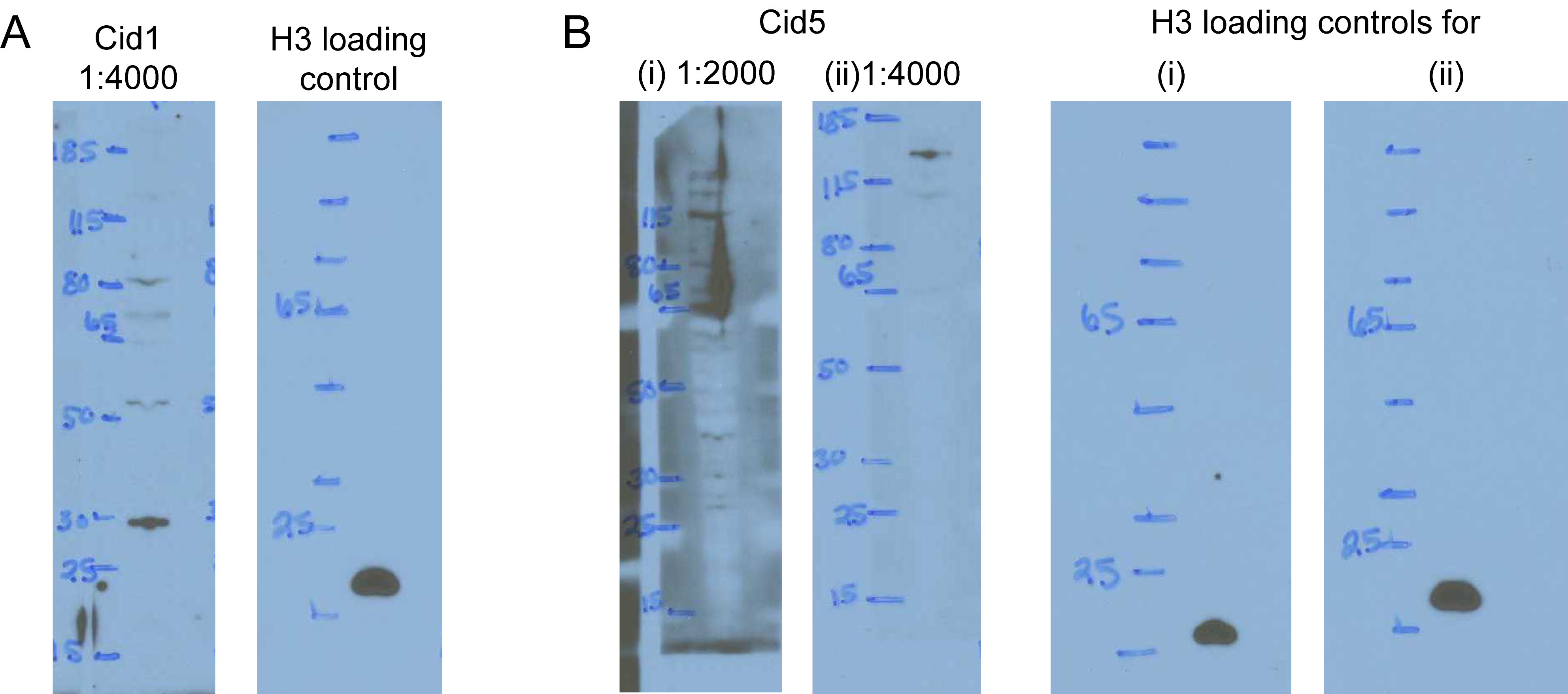

Supplement: Supplementary file 1 [file LSA-2020-00992_SdataF1.tif]
